# The Effect of Solvent Vapor Annealing on Drug-Loaded Electrospun Polymer Fibers

**DOI:** 10.3390/pharmaceutics12020139

**Published:** 2020-02-06

**Authors:** Yu-Jing Chiu, Ziwei Zhang, Karolina Dziemidowicz, Christos-Georgios Nikoletopoulos, Ukrit Angkawinitwong, Jiun-Tai Chen, Gareth R. Williams

**Affiliations:** 1 Department of Applied Chemistry, National Chiao Tung University, Hsinchu 30010, Taiwan; e226690@gmail.com; 2 Sustainable Chemical Science and Technology, Taiwan International Graduate Program, Academia Sinica and National Chiao Tung University, Hsinchu 30010, Taiwan; 3 Center for Emergent Functional Matter Science, National Chiao Tung University, Hsinchu 30010, Taiwan; 4 UCL School of Pharmacy, University College London, 29-39 Brunswick Square, London WC1N 1AX, UK; ziwei.zhang@ucl.ac.uk (Z.Z.); k.dziemidowicz.16@ucl.ac.uk (K.D.); christos-georgios.nikoletopoulos.18@alumni.ucl.ac.uk (C.-G.N.); ukrit.angkawinitwong.11@ucl.ac.uk (U.A.)

**Keywords:** drug delivery system, poly(ε-caprolactone), post-treatment, electrospinning, annealing

## Abstract

Electrospinning has emerged as a powerful strategy to develop controlled release drug delivery systems but the effects of post-fabrication solvent vapor annealing on drug-loaded electrospun fibers have not been explored to date. In this work, electrospun poly(ε-caprolactone) (PCL) fibers loaded with the hydrophobic small-molecule spironolactone (SPL) were explored. Immediately after fabrication, the fibers are smooth and cylindrical. However, during storage the PCL crystallinity in the fibers is observed to increase, demonstrating a lack of stability. When freshly-prepared fibers are annealed with acetone vapor, the amorphous PCL chains recrystallize, resulting in the fiber surfaces becoming wrinkled and yielding shish-kebab like structures. This effect does not arise after the fibers have been aged. SPL is found to be amorphously dispersed in the PCL matrix both immediately after electrospinning and after annealing. In vitro dissolution studies revealed that while the fresh fibers show a rapid burst of SPL release, after annealing more extended release profiles are observed. Both the rate and extent of release can be varied through changing the annealing time. Further, the annealed formulations are shown to be stable upon storage.

## 1. Introduction

The development of new controlled release drug delivery systems (DDSs) remains an important goal in pharmaceutics [1]. Compared with standard immediate release formulations, controlled release has many advantages, for instance permitting the rate and location of drug release to be precisely controlled [2,3,4]. A range of approaches can be used to obtain a controlled release DDS. For example, a reservoir system with a drug-loaded core and polymer coating can be constructed to avoid burst release [5]. Alternatively, the use of thermo- or pH-responsive polymers can allow release to be targeted in vivo [6]. The electrospinning technique is a versatile technology that can be used to prepare a wide range of controlled release DDSs [2].

Electrospinning is a top-down technique that generates polymer fibers with diameters ranging from nanometers to micrometers [7,8]. It does this through the application of electrostatic forces to polymer solutions as they are extruded through a needle. The morphology of the resultant fibers can be adjusted by changing the electrospinning parameters [9]. A large number of studies have reported electrospun polymer fibers with sustained drug release profiles. For example, monoaxial electrospinning has been used to produce nanofibers loaded with nanoparticles and extend drug release times [10]. Engineering the internal fiber architecture can also be used to provide controlled release, such as via coaxial or triaxial electrospinning [11,12,13]. It is even possible to perform “reactive electrospinning” in which polymer crosslinking occurs during the spinning process [14]. However, although electrospun drug delivery systems have been widely investigated, the effects of post-fabrication treatments on their properties have been rarely studied [15].

A range of post-treatment techniques, such as thermal annealing and solvent vapor annealing, have been utilized to adjust the morphologies and properties of polymer materials, including electrospun fibers. When amorphous or semi-crystalline polymer fibers are heated above their glass transition temperatures, the polymer chains can reorient themselves to achieve more stable configurations [16,17]. For instance, in previous work electrospun polystyrene (PS)/poly(methyl methacrylate) (PMMA) core-shell fibers were thermally annealed on PMMA films. The PS cores of the fibers were found to break into a serious of spherical particles with distinct spacings to reduce the surface and interfacial energies [18]. Alternatively, solvent vapor annealing can be applied. This can avoid any polymer decomposition arising from high temperatures, and potentially provides for more controlled annealing [19,20]. Electrospun PS/PMMA core-shell fibers have been annealed with toluene (a good solvent for both PS and PMMA) and cyclohexane (a good solvent for PS but not for PMMA) and the morphologies of the fibers seen to transform to different shapes according to the annealing environment [21,22].

**Figure 1:**
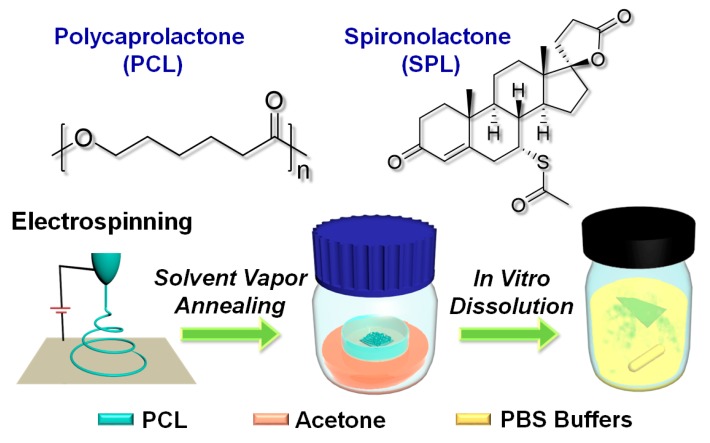
Chemical structures of PCL and SPL, along with a schematic illustration of the experimental procedures adopted in this work.

Poly(ε-caprolactone) (PCL) is explored in this work. PCL is a biodegradable and semi-crystalline polymer which has been widely used for the production of biomedical materials [23,24]. PCL has a crystallization temperature that is similar to ambient temperature (~32 °C [25]). Thus, it is possible that the physical form of the polymer in PCL-based formulations may change under standard storage conditions, and such alterations in crystallinity could affect the performance of a PCL-based drug delivery system. Previously, Liu et al. reported the fabrication of electrospun PCL fibers with secondary nanostructures after annealing with various solvents [16]. During annealing, the amorphous PCL chains are delocalized and redeposited on pre-existing crystalline regions, leading to secondary structures being inscribed onto the fibers [26]. This route could potentially allow the drug release properties of analogous drug-loaded systems to be incrementally varied, in addition to enhancing storage stability. In this study therefore, the effect of solvent vapor annealing on electrospun PCL fibers loaded with the small-molecule drug spironolactone (SPL) is investigated. This is the first study to explore the effect of annealing on electrospun drug delivery systems. A schematic diagram illustrating our approach is presented in Figure 1.

## 2. Materials and Methods

### 2.1. Materials

Polycaprolactone (PCL) (average *M*_n_: 80 kg mol^−1^) was purchased from Sigma Aldrich, Haverhill, UK. Spironolactone (SPL) was acquired from Acros Organics, Loughborough, UK. Dichloromethane (DCM) (≥99.9%) was bought from Merck, Feltham, UK. Dimethylformamide (DMF) was supplied by Tedia, Saint Neots, UK. Acetone (≥99.8%) was obtained from Fisher, Loughborough, UK. Phosphate buffered saline (PBS) (pH value = 7.4) was purchased from Sigma Aldrich, Carlsbad, CA, USA.

### 2.2. Electrospinning

To fabricate the fibers, polymer solutions were first prepared. PCL was dissolved in DCM and DMF (volume ratio of 3:1) at a concentration of 13% *w*/*v*. SPL was then added to the PCL solutions with a concentration of 15% *w*/*w* (with respect to the polymer weight). The solutions were then loaded into a 5 mL plastic syringe (Terumo, Elkton, MD, USA), and a stainless-steel needle with an inner diameter of 0.51 mm (21 gauge, Nordson EFD, Aylesbury, UK) was connected to the syringe. The syringe was mounted on a syringe pump (KDS100, KD Scientific, London, UK). A DC power supply (HCP35-35000, FuG Elektronik, Schechen, Germany) was used to apply a high voltage (10–20 kV) between the needle and a grounded metal collector with a working distance between 10–20 cm. The collector plate was covered with aluminum foil. Electrospinning processes were conducted under ambient conditions (temperature: ~21 °C, relative humidity: ~45%). The optimized electrospinning conditions were as follows: flow rate: 1 mL/h; voltage: 15 kV; working distance: 17 cm. The collected fiber mats were stored at room temperature.

### 2.3. Solvent Vapor Annealing

The fiber mats on aluminum foil were cut into pieces with size of 1×3 cm. These fiber samples were placed in a capless glass sample vial (14 mL). The vial was then placed into a sealed glass serum bottle (100 mL) which contained 40 mL of acetone. The annealing processes were carried out at ambient temperature (~21 °C). After the desired annealing time, the samples were removed and dried at room temperature (~21 °C) to evaporate any residual solvent. Full details of the experimental parameters are listed in Table 1.

### 2.4. Drug Loading and In Vitro Drug Release

The drug content of the fibers was determined by dissolving the as-spun material into DCM and employing UV spectroscopy (Cary 100 UV–Vis instrument, Agilent, Santa Clara, CA, USA). Drug encapsulation efficiency was calculated as a percentage in terms of the actual drug loading divided by the theoretical content. Samples of the SPL-loaded PCL fiber mats (~2 mg) were immersed in 20 mL of PBS (pH 7.4). Dissolution experiments were carried out at 37 °C and with shaking at 100 rpm in a shaker incubator. At predetermined timepoints, 200 μL of the sample medium was withdrawn and stored in an Eppendorf tube (0.5 mL), and an equal amount of preheated PBS buffer added to maintain a constant volume. The release of SPL was quantified at 237 nm by UV spectroscopy. Triplicate experiments were performed, and the results are given as mean ± S.D.

### 2.5. Structure Analysis and Characterization

The morphologies of the samples were examined by scanning electron microscopy (SEM) on a Quanta 200F instrument (FEI, Hillsboro, OR, USA) at an acceleration voltage of 10 kV. Before SEM imaging, the samples were coated with gold for 60 s. Fiber diameters were measured using Photoshop (CS5, Adobe, San Jose, CA, USA), and the mean calculated from more than 100 measurements.

Fourier-transform infrared spectroscopy was performed on a Spectrum 100 instrument (PerkinElmer, Waltham, MA, USA) in attenuated total reflectance mode. Samples were scanned over the range of 4000 to 650 cm^−1^ with the resolution set at 1 cm^−1^, and 20 spectra collected per sample.

XRD patterns were obtained on a Miniflex 600 instrument supplied with Cu Kα radiation (Rigaku, Tokyo, Japan; λ = 1.5418 Å) at a voltage of 40 kV and current of 15 mA. Measurements were recorded over the 2θ range of 3–60° (scan speed: 5° min^−1^). DSC measurements were collected on a Q2000 instrument (TA Instruments, New Castle, DE, USA). Samples were sealed in aluminum pans (Tzero pan and lid, TA Instruments, New Castle, DE, USA) and heated from 0 to 250 °C at 10 °C min^−1^ under a nitrogen purge of 50 mL min^−1^. The TA Instruments Universal Analysis software was used to analyze the data. To calculate the crystallinity of the samples, the measured values of enthalpy were divided by the melting enthalpy for 100% crystalline PCL (139.5 J g^−1^) [27].

X-ray photoelectron spectroscopy (XPS) measurements were acquired with a K-Alpha X-Ray system (ThermoFisher Scientific, Waltham, MA, USA). This is equipped with an aluminum K-alpha micro-focused monochromator source (1486.68 eV) of 100 W, and measurements were taken at a chamber pressure below 2 × 10^−8^ Torr. Total scan numbers of 15 were used to survey C_1s_, O_1s_, and S_2p_.

## 3. Results and Discussion

### 3.1. Fiber Fabrication

Figure 1 presents the molecular structures of PCL and SPL and a schematic illustration of the experimental processes. Both PCL and SPL are hydrophobic and should be highly compatible with one other [28]. Electrospinning was first used to prepare PCL and SPL-loaded PCL fibers. Subsequently, the fibers were annealed with acetone vapor for varied times. This resulted in eight samples, detailed in Table 1.

**Figure 2:**
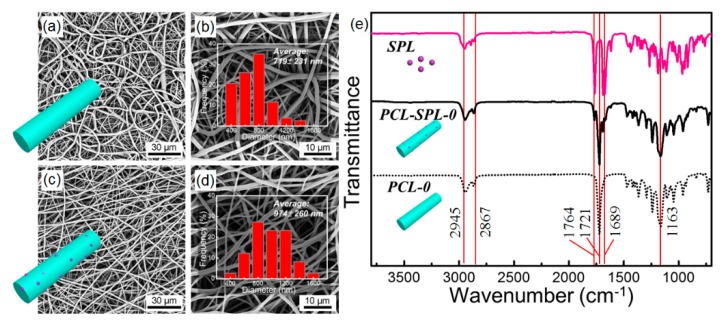
SEM images and diameter distributions for the fresh (**a**,**b**) PCL and (**c**,**d**) SPL-loaded PCL fibers. (**e**) FT–IR spectra.

Figure 2a–d show SEM images of the products obtained by electrospinning. Smooth cylindrical fibers were produced after optimization, with the optimal processing parameters being a flow rate of 1 mL/h, applied voltage of 15 kV, and working distance of 17 cm. From Figure 2a–d respectively, it can be seen that the morphology of the fresh PCL (PCL-0) and the SPL-loaded fibers (PCL-SPL-0) are similar, and the average diameters are 719 ± 231 nm (Figure 2b) and 974 ± 260 nm (Figure 2d) respectively. The larger diameter of the PCL-SPL-0 fibers can be attributed to the greater mass of solute dispensed per unit time.

The loading of SPL in the PCL-SPL-0 fibers can be confirmed by FT–IR spectroscopy (Figure 2e). For PCL-0, the characteristic peaks from ether (C-O-C) and carbonyl (C=O) stretching appear at 1163 and 1721 cm^-1^ [29]. For SPL, characteristic peaks from thioacetyl and γ-lactone stretching appear at 1689 and 1764 cm^−1^. Bands at 2867 and 2945 cm^−1^ correspond to symmetric and asymmetric CH_2_ stretching [30]. In the FT–IR spectrum of PCL-SPL-0 the characteristic peaks of both PCL and SPL are observed, confirming the presence of SPL in the fibers.

**Figure 3:**
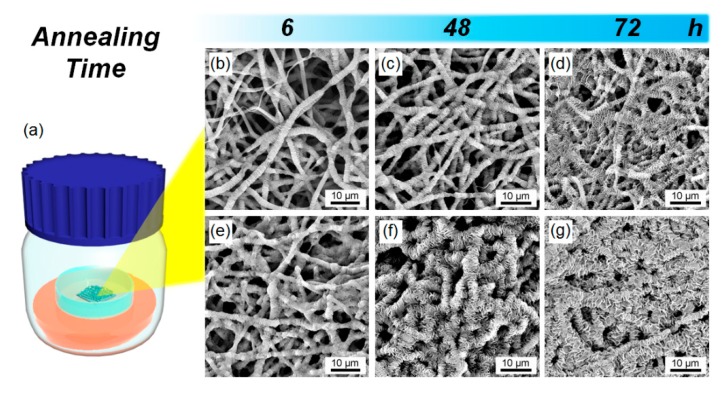
(**a**) Graphical illustration of the solvent vapor annealing process. (**b**–**g**) SEM images of (**b**) PCL-6, (**c**) PCL-48, (**d**) PCL-72, (**e**) PCL-SPL-6, (**f**) PCL-SPL-48, and (**g**) PCL-SPL-72.

Solvent vapor annealing was applied to freshly collected electrospun fibers. Figure 3a depicts an illustration of the solvent vapor annealing process. The electrospun fiber mats were cut into pieces and put inside a glass sample vial. The vial was then placed into a sealed acetone-containing bottle. PCL and SPL will both dissolve slightly in this environment, leading to molecular rearrangement.

SEM images of the annealed fibers are shown in Figure 3b–g. It can be seen that the surface roughness of the fibers increases significantly with the annealing time, resulting in shish-kebab like structures. This arises because the amorphous PCL chains have more time to delocalize and recrystallize. The annealed fibers were examined by FT–IR spectroscopy, and the results are summarized in Figure S1 (Supplementary Information). The spectra are essentially identical before and after annealing, confirming that there is no change in the chemical structure during this process. These findings all concur with previous studies applying annealing to electrospun fibers, including those by Liu et al. [16] and Bauer et al. [26].

### 3.2. Aging

PCL is a semicrystalline polymer, which exhibits a glass transition temperature (*T*_g_), crystallization temperature (*T*_c_), and melting point (*T*_m_) of approximately −61, 32, and 60 °C, respectively [25]. When the fibers are stored at ambient temperature (~21 °C), above the *T*_g_, it is possible that the fibers will undergo some level of molecular rearrangement upon aging. To explore this, the fibers were aged for different periods of time (4 and 40 days; see Figure S2 and Table S1). Differential scanning calorimetry (DSC) was used to quantify the crystallinity of the samples, and the results are given in Figure S2a and Table S2. Figure S2b plots fiber crystallinity against aging time. With longer aging times, the crystallinity of the fibers increases. Both the PCL and SPL-loaded fibers show the same trend.

To study how the aging process affects annealing, the fibers were aged for 4 and 30 d and then annealed for 48 h in acetone vapor. Figure S3a–c present the SEM images of the resultant fibers. For fibers which are annealed immediately after fabrication, shish-kebab structures are formed (Figure S3a). However, if the fibers are aged at ambient temperature and then annealed, they retain their initial smooth surfaces (Figure S3b,c). A possible explanation for this might be that some parts of the amorphous PCL chains become fixed during aging. The calculated crystallinity percentages (Table S3) are all similar regardless of aging time, which might be because all of the samples were annealed for the same period of time. To make sure annealing was effective, all annealing was thus conducted immediately after electrospinning.

### 3.3. Physical Form Characterisation

**Figure 4:**
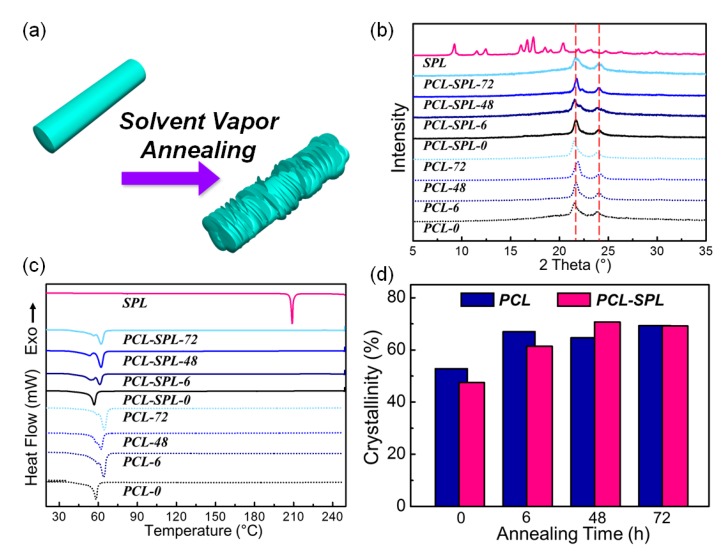
(**a**) Graphical illustration of the fibers before and after annealing. (**b**) XRD patterns. (**c**) DSC curves (exo up). (**d**) Plot of percentage crystallinity against the annealing time.

After annealing with acetone for different periods of time, the smooth surface of the fibers becomes wrinkled, likely owing to recrystallization of the amorphous PCL chains on the crystalline PCL parts, as illustrated in Figure 4a. To see how the interactions between SPL and PCL are affected X-ray diffraction (XRD) and DSC data were collected. Figure 4b depicts the XRD patterns for the fibers. All show the major reflections from semi-crystalline PCL (at 2θ of 21° and 23.5°) [31,32]. It can be inferred that SPL is amorphously dispersed in the PCL fiber matrices because its major Bragg reflections (9.1, 11.5, 12.5, 16.3, 19.1, 17.5, 18.8, 19.2, 20.21, and 23.1°) are not present in the fibers’ patterns [33].

The DSC data (Figure 4c) reveal that the melting temperature for SPL is ~210 °C, and for PCL is ~60 °C, in agreement with the literature [24,34]. The thermograms from the fibers do not show any melting events from SPL, confirming that the drug is amorphously dispersed in the fibers. All do, however, show the PCL melting endotherm at ~60 °C. After integration of the peaks, the melting enthalpies were determined and are summarized in Figure 4d (full details are in Table S4). For both the PCL (blue bars) and SPL-loaded (pink bars) fibers, similar trends can be seen: with longer annealing times, the percentage of crystalline material increases. It can also be seen that double melting points appeared in some annealed samples, which might arise from the presence of both the original and secondary crystalline structures [16].

### 3.4. Drug Loading and In Vitro Release

To determine drug loading, the fibers were dissolved in dichloromethane and analysed using UV spectroscopy (Figure S4). The drug loading is found to be 13.0 ± 0.0% and the encapsulation efficiency 86.4% for the PCL-SPL-0 fibers, indicating that the majority of the drug in the feedstock is carried through into the fibers. Given the hydrophobic nature of SPL (solubility in water: ca. 30 µg/mL^−1^ [30]), it is thought the residual must have adsorbed to the plasticware used during electrospinning.

**Figure 5:**
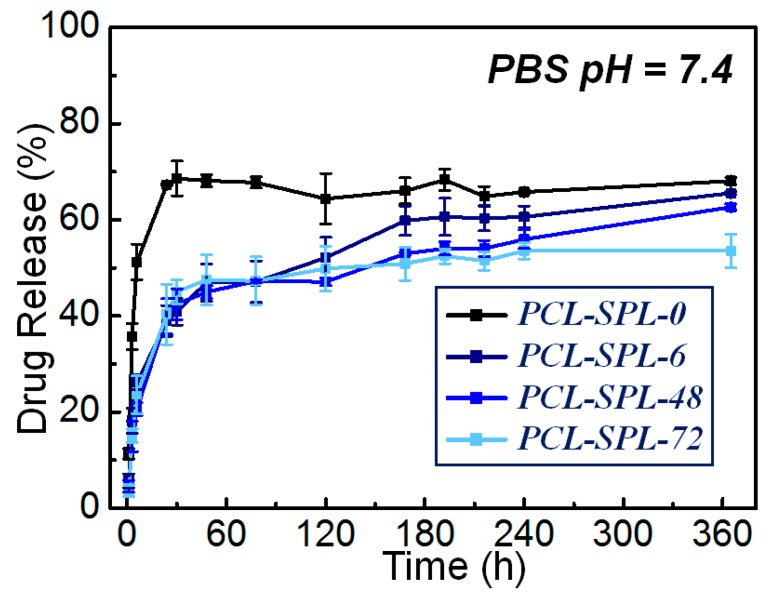
In vitro SPL release profiles. Data are represented as mean ± S.D. from three independent experiments.

In vitro dissolution studies were performed to elucidate the effect of annealing on drug release. The fiber mats were immersed in phosphate buffered saline (PBS) (pH = 7.4) at 37 °C with shaking at 100 rpm. A UV standard curve for SPL was constructed (Figure S5) and used to quantify drug release. The release profiles are given in Figure 5. The non-annealed PCL-SPL-0 fibers show a burst of release in the first 30 h, reaching ca. 70% release. There is then no further release from this system. The annealed fibers show rather different release profiles. Initially, there is a burst of release in the first 30 h, and then an extended period of release. It can be seen that for the annealed SPL-loaded PCL fibers, approximately 50% of SPL is released after 60 h, a lower value than for the non-annealed PCL-SPL-0 system. The additional crystallinity introduced by the annealing process thus delays the drug release process, and after 360 h the overall release percentages lie in the order PLC-SPL-0 > PCL-SPL-6 > PCL-SPL-48 > PCL-SPL-72. A longer annealing time hence further retards drug release.

The Ritger–Peppas model was applied to analyze the release mechanism of the fibers, and the results are summarized in Table S5 and Figure S6. The Ritger-Peppas model is given in Equation (1).

(1) $$\frac{M_{t}}{M_{\infty }}=kt^{n}$$

*k* is a rate constant, *M*_t_*/M*_∞_ is the fraction of drug released at time *t,* and *n* is the Peppas exponent [35]. It can be seen (Table S5) that the n values for all the formulations are less than 0.5, which implies the rate limiting step to release is the diffusion of SPL through the polymer matrix [36].

To compare the dissolution profiles, F2 factors were calculated based on Equation (2):

(2) $$F2\;=\;50\times \log\left\{{\left[1+\frac{1}{n}\overset{t=n}{\underset{t=1}{\sum }}{\left(R_{t}-T_{t}\right)}^{2}\right]}^{-0.5}\times 100\right\}$$

*R_t_* and *T_t_* are the cumulative percentage release at time *t* of the reference and the test sample, respectively [37]. The F2 values for PCL-SPL-0 compared to the fibers annealed for 6, 48, and 72 h are 38, 36, and 36, respectively. An F2 value of 50–100 indicates that the two dissolution profiles are similar. It is, thus, clear that the annealed fibers have different release profiles to the as-prepared formulation.

### 3.5. Annealing and Release Mechanisms

The fibers were recovered at the end of the in vitro studies and imaged by SEM (Figure S7). It can be seen that the fiber morphology is unchanged after immersion in PBS for 360 h. The non-annealed fibers remain cylindrical with smooth surfaces, while the annealed fibers retain the shish-kebab structures visible in the SEM images shown in Figure 3.

**Figure 6:**
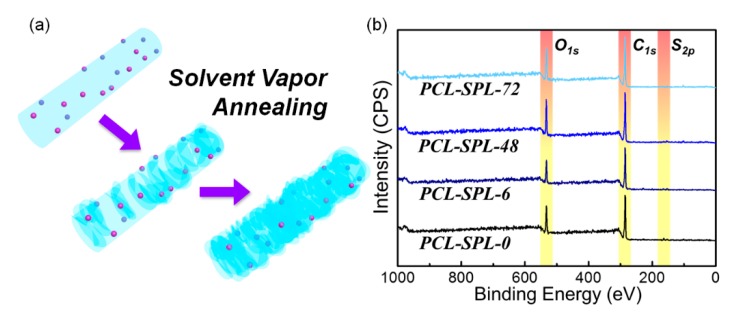
(**a**) Schematic illustration of the transformation process to form shish-kebab structures. (**b**) XPS spectra of the SPL-loaded fibers.

The proposed formation mechanism of the shish-kebab structures during annealing is depicted in Figure 6a. Throughout the annealing process, the amorphous PCL chains are swollen by acetone vapor. With longer periods of annealing, the amorphous parts begin to recrystallize on the crystalline parts, forming the shish-kebab structures and transforming the fiber surfaces from smooth to wrinkled. During the recrystallization process, the amorphous PCL chains are rearranged, and thus it is possible that the SPL molecular distribution in the PCL matrix could change.

To analyze the surface elements of the formulations, X-ray photoelectron spectroscopy (XPS) measurements were conducted (Figure 6b). Because SPL contains sulfur, the S_2p_ peak can be regarded as a marker of the drug distribution at the fiber surfaces. The surface C, O, and S contents are summarized in Table S6. It can be seen the percentage of S at the surface declines with annealing time. This is consistent with the recrystallization of the PCL on the fiber surface causing the SPL to become covered and embedded in the fibers (Figure 6a). This results in both slower drug release and a reduced final release percentage.

### 3.6. Stability

It is shown above that PCL-based formulations aged at room temperature are not susceptible to annealing. To investigate the stability of the annealed fibers, the formulations were stored at ambient conditions (temperature: ~21 °C, relative humidity: ~45%) for two months, and the results are shown in Figure S8. The DSC curves of the aged materials (Figure S8a) show the PCL melting point, as expected. No SPL melt is visible, demonstrating that SPL remains amorphously distributed in the fibers. The percentage PCL crystallinity was calculated and is summarized in Table S7. Figure S8b depicts a comparison between fresh formulations and those stored for two months. For the formulations without annealing, the percentage of PCL crystallinity increases after storage for two months, as a result of the movements of the amorphous polymer chains. For the annealed formulations, the PCL crystallinity is similar before and after aging. It can thus be inferred that annealing can stabilize the formulations to aging, and reduce the possibility for performance degradation after storage.

## 4. Conclusions

Electrospun PCL and SPL-loaded PCL nanofibers were fabricated and annealed with acetone vapor. After annealing, the formulations transformed from having cylindrical morphologies to fibers with wrinkled surfaces resembling shish kebabs. This effect only arises if the fibers are annealed immediately after production, and not if they are first aged. XRD and DSC showed that SPL was amorphously dispersed in the PCL matrix both before and after annealing. The PCL crystallinity increased with longer annealing times.

In vitro dissolution tests showed that the annealed formulations led to more extended release profiles, while the fresh fibers gave a burst release of 70% within 30 h. Slower SPL release (over up to 360 h), and a reduced final SPL release percentage (ca. 50–60%) can be observed with the annealed fibers. This is a result of the SPL becoming embedded inside the PCL matrix after annealing, as was demonstrated from XPS experiments. The annealed formulations do not change with aging at room temperature, while non-annealed analogues are found to exhibit PCL recrystallisation upon storage. This work thus shows for the first time that post-fabrication annealing is a potent route to both tune the release profile and enhance the storage stability of electrospun fibers. It is to be expected that this annealing method could potentially be applied to other semi-crystalline polymers and/or those which show relaxation of their amorphous forms. There are a wealth of such polymers used in pharmaceutical applications, including poly(lactic acid), poly(lactic-*co*-glycolid acid) and poly(ethylene oxide), and the solvent annealing route could thus offer a suitable approach to tune the properties of electrospun biomedical materials.

## Figures and Tables

**Table 1:** Details of the formulations reported in this work. All solutions were dissolved in dichloromethane (DCM) and dimethylformamide (DMF; volume ratio = 3:1).

Sample Name	Polymer Conc. (% *w*/*v*)	Drug Loading Conc. (% *w*/*w*) ^a^	Annealing Time (h)
PCL-0	13	--	--
PCL-6	13	--	6
PCL-48	13	--	48
PCL-72	13	--	72
PCL-SPL-0	13	15	--
PCL-SPL-6	13	15	6
PCL-SPL-48	13	15	48
PCL-SPL-72	13	15	72

^a^ with respect to the polymer concentration.

## Supplementary Materials

The following are available online at https://www.mdpi.com/1999-4923/12/2/139/s1, Table S1: sample details for aging experiments, Table S2: *T*_g_, *T*_m_, enthalpy of fusion, and percentage crystallinity for aged samples, Table S3: *T*_m_, fusion enthalpy, and percentage crystallinity for the aged and annealed fibers, Table S4: *T*_m_, fusion enthalpy, and percentage crystallinity for PCL fibers and SPL-loaded PCL fibers after different annealing times, Table S5: the results of fitting the Ritger-Peppas models. Table S6: atomic ratios for the annealed SPL-loaded fibers, Table S7: *T*_m_, fusion enthalpy, and percentage crystallinity of annealed fibers stored over 2 months, Figure S1: FT–IR spectra of the annealed fibers, Figure S2: DSC curves and crystallinity of samples after varied aging times, Figure S3: SEM images and DSC curves of fibers aged for different time and then annealed, Figure S4: Standard curve of SPL in dichloromethane, Figure S5: Standard curve of SPL in PBS at pH 7.4, Figure S6: Fits of the Ritger-Peppas models for annealed fibers, Figure S7: SEM images of annealed fibers after drug release, Figure S8: the results of stability studies after storage of the annealed fibers for 2 months.
