# Supplementary material for: The Effect of Solvent Vapor Annealing on Drug-Loaded Electrospun Polymer Fibers
Source: Pharmaceutics. 2020 Feb 6;12(2):139. doi: 10.3390/pharmaceutics12020139 (PMC7076406; doi:10.3390/pharmaceutics12020139)
Supplement: Supplementary file 1 [file pharmaceutics-12-00139-s001.pdf]

pharmaceutics

Supplementary Materials: The Effect of Solvent Vapor Annealing on Drug-Loaded Electrospun Polymer Fibers

Yu-Jing Chiu, Ziwei Zhang, Karolina Dziemidowicz, Christos-Georgios Nikoletopoulos, Ukrit Angkawinitwong, Jiun-Tai Chen * and Gareth R. Williams *

Table S1. Details of the samples explored in aging experiments.

Sample Name	Polymer conc. (% w/v)	Drug Loading conc. (% w/w)	Aging Time (d)
PCL	13	--	0
PCL-A4	13	--	4
PCL-A40	13	--	40
PCL-SPL	13	15	0
PCL-SPL-A4	13	15	4
PCL-SPL-A40	13	15	40

Table S2. T_g, T_m, enthalpy of fusion, and percentage crystallinity for fresh samples and those aged for 4 and 40 days (denoted A4 and A40, respectively).

Sample Name	Glass Transition Temperature (T_g) (°C)	Melting Point (T_m) (°C)	Enthalpy (J/g)	Crystallinity (%)
PCL	-62.4	52.6±0.5	69.5±1.4	49.8±0.9
PCL-A4	-63.9	53.6±0.6	70.5±5.9	50.5±4.2
PCL-A40	-65.2	55.6±0.1	73.9±3.1	53.0±2.2
PCL-SPL	-70.8	47.4±0.3	59.7±2.9	42.8±2.1
PCL-SPL-A4	-68.5	49.6±0.8	61.3±3.8	43.9±2.7
PCL-SPL-A40	-70.1	54.5±0.3	65.9±2.9	47.2±2.1

Table S3. Table of the T_m, fusion enthalpy, and percentage crystallinity for the aged and annealed fibers.

Sample Name	Melting Point (T_m) (°C)	Enthalpy (J/g)	Crystallinity (%)
PCL-48	57.25	81.50	58.42
PCL-A4-48	55.41	82.73	59.30
PCL-A30-48	55.70	72.79	55.16

Table S4. Table of the T_m, fusion enthalpy, and percentage crystallinity for PCL and SPL-loaded PCL fibers after different annealing times.

Sample Name	Melting Point (T_m) (°C)	Enthalpy (J/g)	Crystallinity (%)
PCL-0	55.1	73.7	52.8
PCL-6	60.0	93.5	66.9
PCL-48	60.5	90.2	64.7
PCL-72	60.6	96.7	69.3
PCL-SPL-0	53.6	66.2	47.5
PCL-SPL-6	57.6	85.7	61.4
PCL-SPL-48	58.2	98.6	70.7
PCL-SPL-72	57.9	96.5	69.2

Table S5. The results of fitting the Ritger-Peppas model to SPL release from the fiber formulations.

Value	PCL-SPL-0	PCL-SPL-6	PCL-SPL-48	PCL-SPL-72
k (h^{-1})	0.357	0.216	0.243	0.354
n	0.13	0.19	0.15	0.077
R^2	0.59	0.95	0.91	0.93

Table S6. Atomic ratios for the annealed SPL-loaded fibers, as determined by XPS.

Sample Name	C_{1s} (%)	O_{1s} (%)	S_{2p} (%)
PCL-SPL-0	81.4±1.8	17.6±1.2	0.9±0.7
PCL-SPL-6	82.6±1.2	16.7±1.1	0.6±0.8
PCL-SPL-48	80.1±1.5	19.5±2.2	0.4±0.8
PCL-SPL-72	80.6±0.9	19.4±0.9	0.0±0.0

Table S7. T_m, fusion enthalpy, and percentage crystallinity of annealed fibers subjected to aging at room temperature for two months.

Sample Name	Melting Point (T_m) (°C)	Enthalpy (J/g)	Crystallinity (%)
PCL-SPL-0	54.8	74.6	53.5
PCL-SPL-6	58.7	84.3	60.4
PCL-SPL-48	60.7	96.8	69.4
PCL-SPL-72	60.1	99.4	71.3

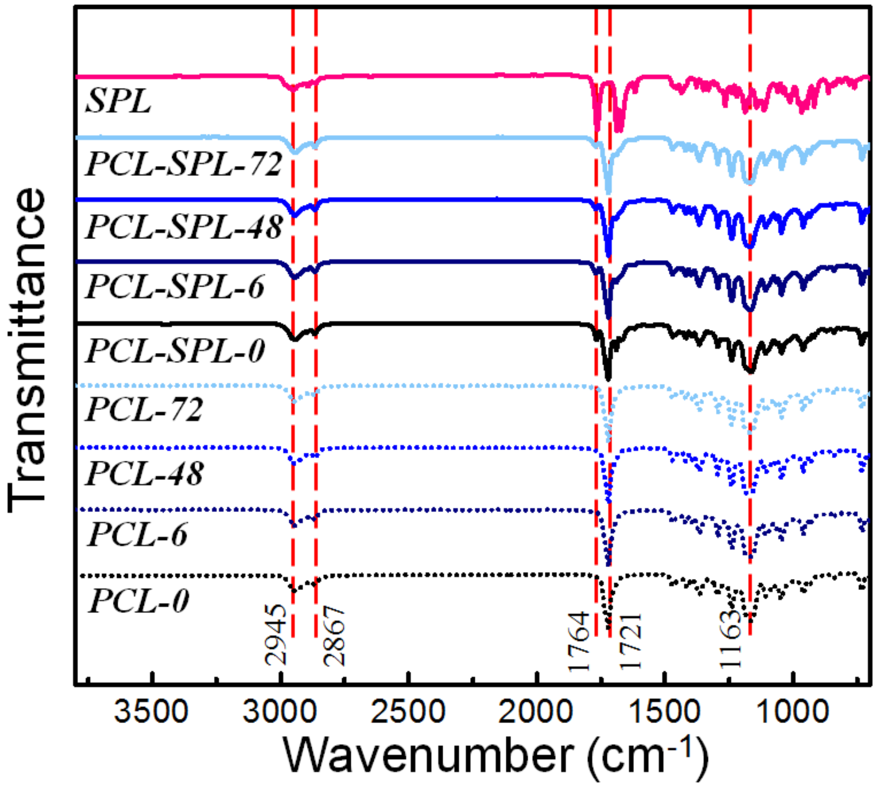

Figure S1. FT-IR spectra of the annealed fibers.

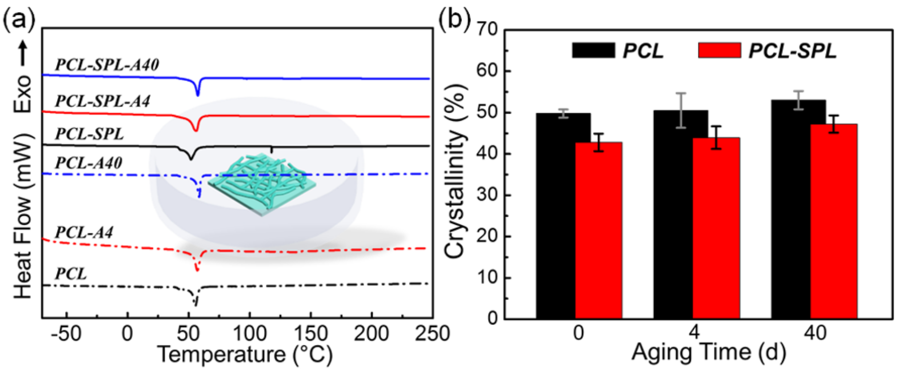

Figure S2. (a) DSC curves (exo up) and (b) PCL crystallinity of samples for fresh samples and those aged for 4 and 40 days (denoted A4 and A40, respectively).

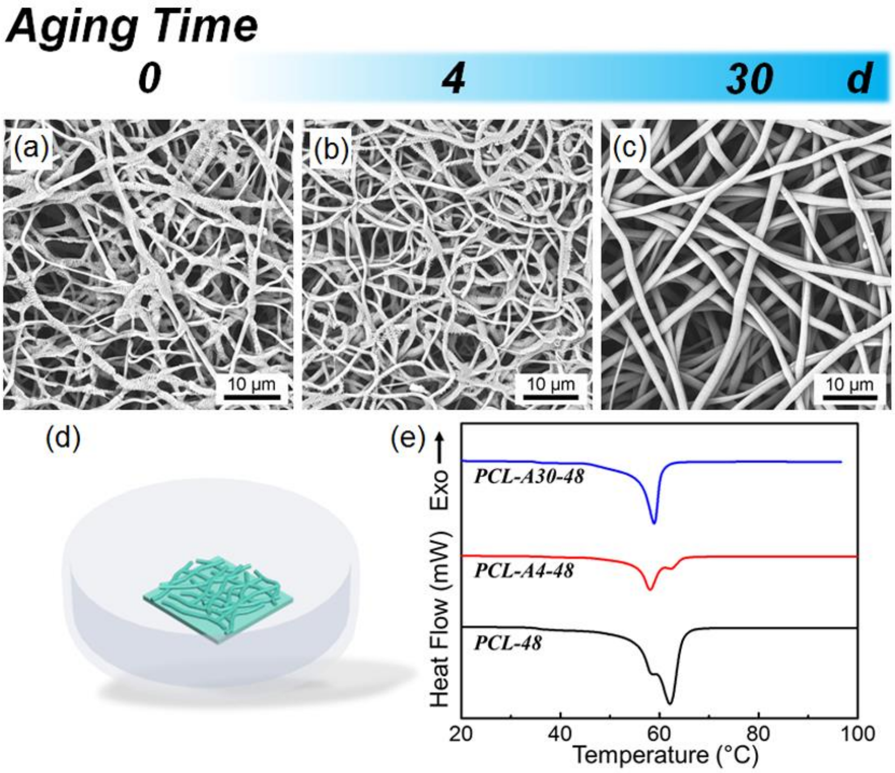

Figure S3. (a-c) SEM images of PCL fibers aged for 0, 4, and 30 days and then annealed with acetone for 48 h. (d) Graphical illustration of aging of the fibers at room temperature. (e) DSC curves (exo up).

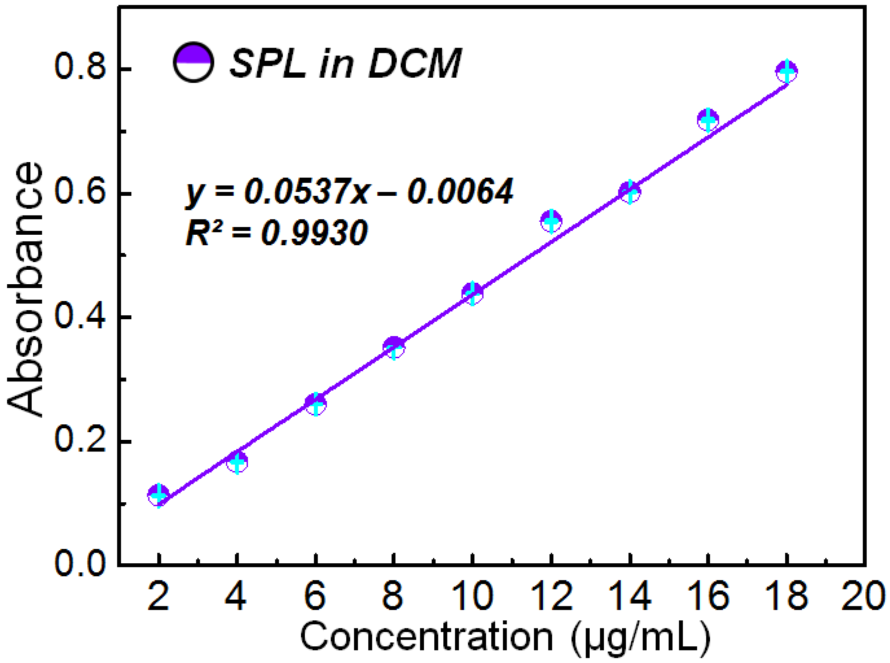

Figure S4. Standard curve of SPL in dichloromethane.

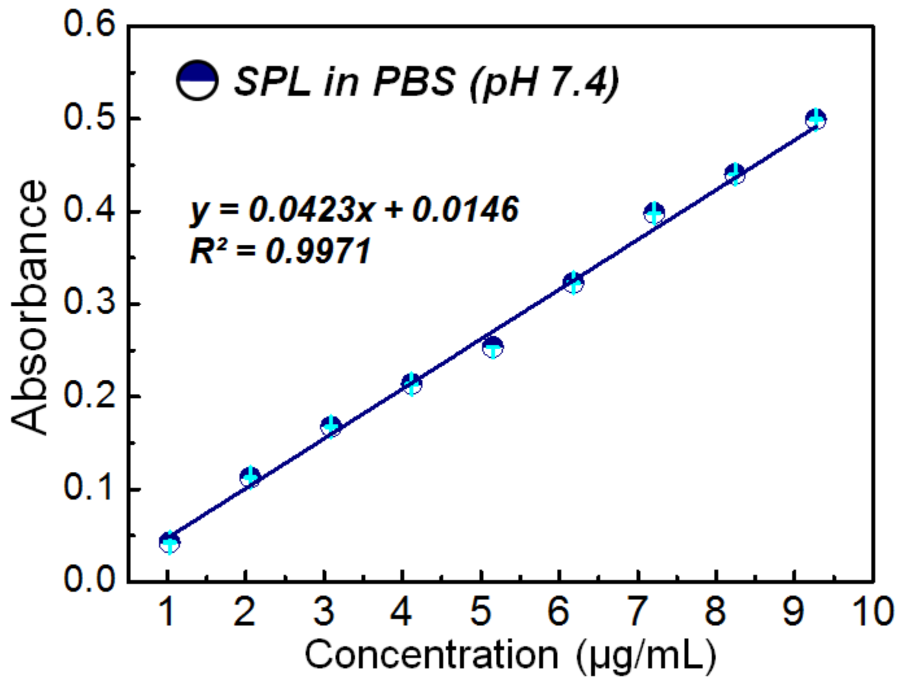

Figure S5. Standard curve of SPL in PBS at pH 7.4.

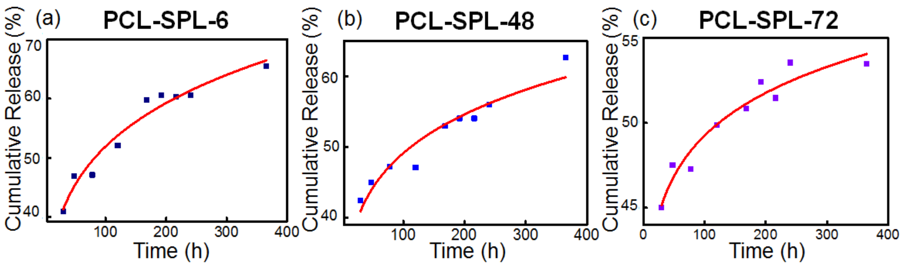

Figure S6. Fits of the Ritger-Peppas models to the release data for (a) PCL-SPL-6, (b) PCL-SPL-48, and (c) PCL-SPL-72 fibers from 0 to 360 h.

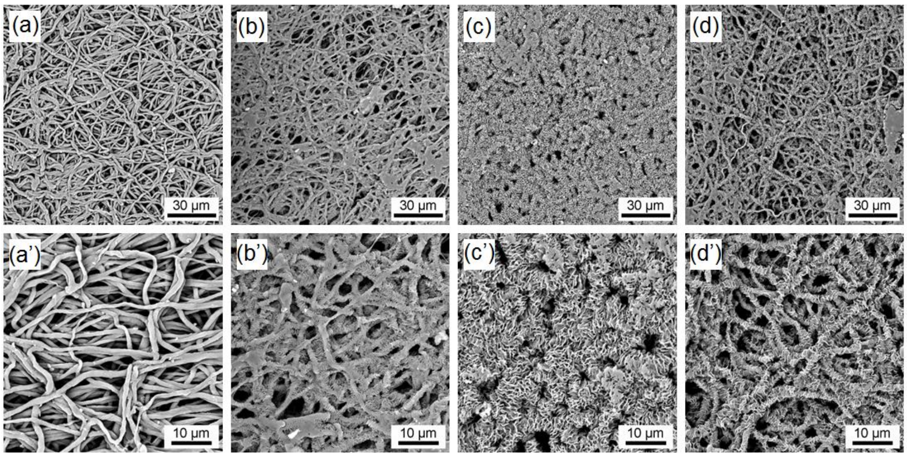

Figure S7. SEM images with (a-d) lower and (a'-d') higher magnifications of the SPL-loaded PCL fibers after 15 days' immersion in PBS. Images are shown for (a,a') PCL-SPL-0, (b,b') PCL-SPL-6, (c,c') PCL-SPL-48, and (d,d') PCL-SPL-72.

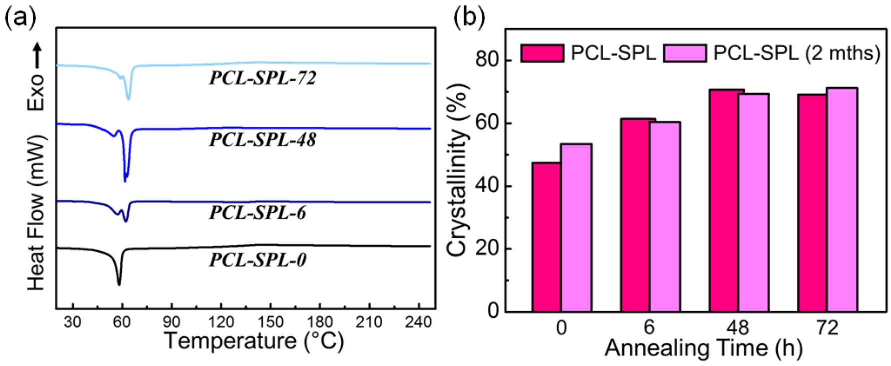

Figure S8. The results of stability studies after storage of the annealed fibers at ambient temperature for two months. (a) DSC curves (exo up). (b) Plot of the percentage of PCL crystallinity against the annealing time for annealed SPL-loaded fibers, both immediately after annealing and after two months' storage.
